# Supplementary figures and images for: Retrospective Observational Study to Determine the Epidemiology and Treatment Patterns of Patients with Triple-Negative Breast Cancer
Source: Cancers (Basel). 2024 Mar 7;16(6):1087. doi: 10.3390/cancers16061087 (PMC10968507; doi:10.3390/cancers16061087)

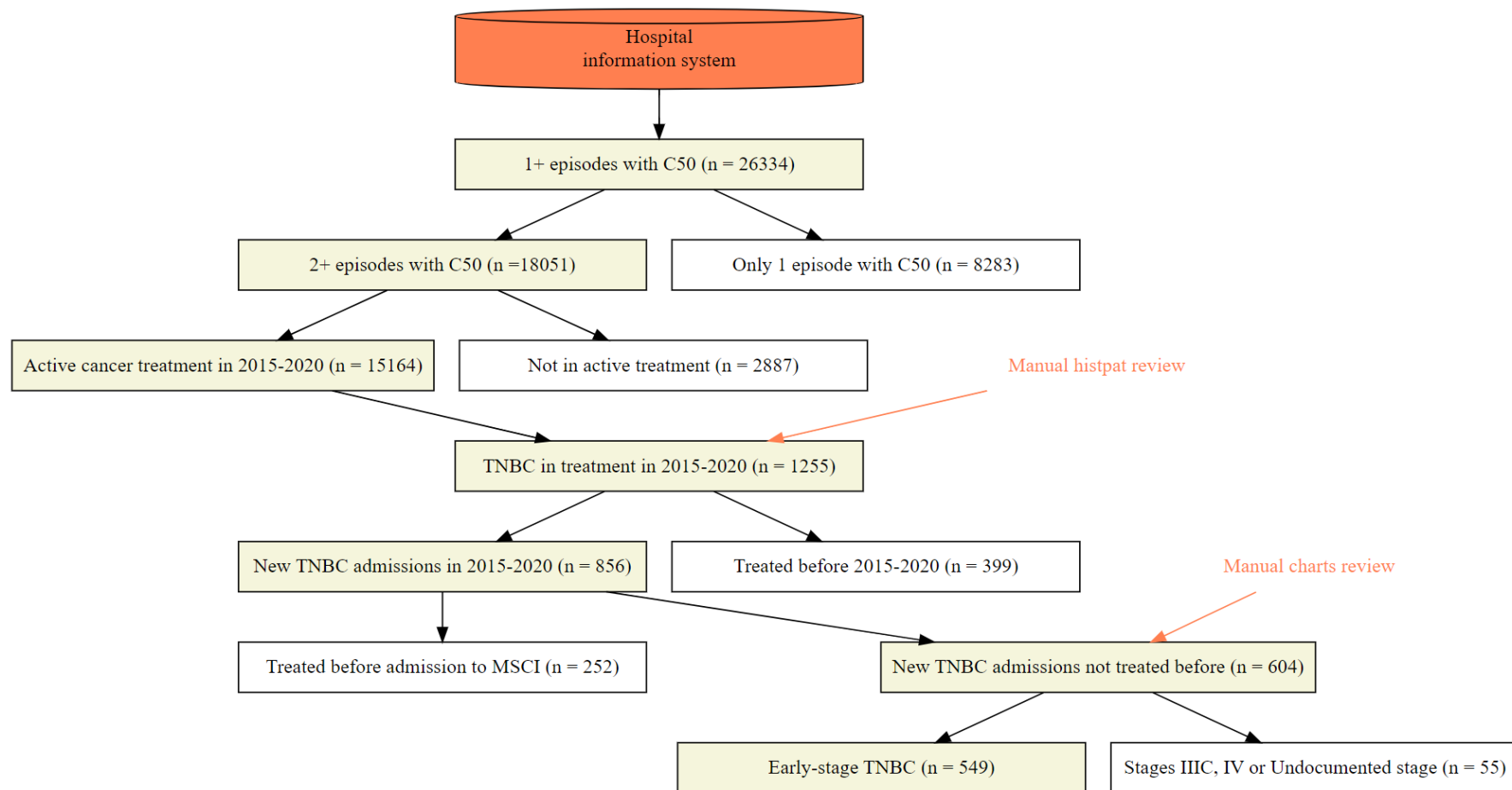

**Figure S1.** Scheme of the study sample selection.

Supplement: Supplementary file 1 [file cancers-16-01087-s001.zip › cancers-2893564-supplementary Figure S1.pdf]
